# Mr. Woodward, on Infantile Diseases

**Published:** 1800-07

**Authors:** W. Woodward


### *To the Editors of the Medical and Phyſical Journal.*

Gentlemen,

If you think the following Eſſay has ſufficient merit to be admitted into your uſeful Publication, I will thank you to inſert it.

Your's, &c.

*May* 22, 1800. W. WOODWARD.

---

By the moſt accurate calculation,* half of mankind die under eight years of age. The extraordinary havock made by diſeaſes among children, is much owing to the unnatural treatment they meet with, which is ill ſuited to the ſingular delicacy of their tender frames. This mortality is greateſt among the moſt luxurious part of mankind, but gradually decreaſes in proportion as the diet becomes ſimpler, the exerciſe more frequent, and the general method of living more hardy; and as it doth not take place among wild animals, the general foundation of the cauſe is ſufficiently pointed out. The inſtincts of children, and the conduct of Nature in rearing other animals, are never attended to, and they are incapable of helping themſelves, particularly in the moſt early period. When they are farther advanced in life, the voice of Nature becomes too loud to be ſtifled; and then, in ſpite of the influence of corrupted and adventitious taſte, will be obeyed.

Many reaſons have been aſſigned why the ſtate of infancy is the moſt ſickly; and why ſo great a proportion of the human ſpecies is cut off at that early period. Phyſicians have inſiſted largely on the unavoidable dangers ariſing from the ſudden

* Since the practice of inoculation has become ſo general, this may have ſomewhat decreaſed; and the introduction of the vaccine diſeaſe bids fair even to leſſen that: To the honour of thoſe gentlemen, who, in the cauſe of humanity, brought this diſeaſe forward, be it ever remembered.

ſudden and total change of the animal œconomy of infants, that commences immediately on their birth; and on the dangers that are cauſed by the free admiſſion of the external air to their bodies at that time: they have expatiated on the high degree of irritability of their nervous ſyſtem, the delicacy of their whole frame, and the acceſſency of their food. A little reflection, however, may ſhew us, that this account of the matter, though plauſible at firſt view, is not ſatisfactory: This ſingle conſideration refutes it, that all theſe alleged cauſes of the ſickneſs of infants are not peculiar to the human ſpecies, but are found among many other animals, without being attended by ſuch effects; that the diſeaſes moſt common to children are not found among the ſavage part of mankind; and that they prevail in exact proportion to the effeminacy and luxury, and as people forſake the plain dictates of Nature, to follow what they are pleaſed to term, *the light of reaſon.* There is, in truth, a greater luxuriancy of life and health in infancy, than in any other period of life. Infants, we acknowledge, are more delicately ſenſible to injury than thoſe in advanced life; but to compenſate this, their fibres and veſſels are more capable of diſtenſion, their whole ſyſtem is more flexible, their fluids are leſs acrid, and leſs diſpoſed to putreſſence; they bear all evacuations more eaſily, except that of blood; and, which is an important circumſtance in their favour, they never ſuffer from the terrors of a diſtracted imagination: their ſpirits are lively and equal; they quickly forget their paſt ſufferings, and never anticipate the future.

Children recover from diſeaſes under ſuch unfavourable circumſtances as are never ſurvived by adults; if they waſte more quickly under ſickneſs, their recovery is quick in proportion, and generally more compleat than in older people, as diſeaſes ſeldom leave thoſe baneful effects on their conſtitutions, ſo frequent in thoſe of adults: in ſhort, a phyſician ought never to deſpair of a child's life while it continues to breathe.

In human parturition, the moſt known and ſucceſsful practitioners, if they are candid, will own, that in common and natural caſes, Nature is ſufficient; and, that their buſineſs is only to aſſiſt her efforts in caſes of weakneſs of the mother, or an unnatural poſition of the child. As ſoon as an infant comes into the world, our firſt care is to cram it with phyſic: There is a liquor in the bowels of infants, and many other animals, when they are born, which is neceſſary to be carried off; the medicine which Nature has provided for that purpoſe, is the mother's firſt milk; this, indeed, anſwers every purpoſe, and effectually; but we think ſome drugs forced down the child's throat will do much better—the compoſition of which varies,

 according according to the fancy of the good woman who presides at the birth. It deserves to be remarked, while on that subject, that calves, which are the only animals taken under our peculiar care in these circumstances, are treated in the same manner; they have the same sort of physic administered to them, and often with the same success; many dying under the operation, or of its consequences: and we have the greatest reason to think, that more of that species of animals die at this period than of all others. We see in these circumstances put together, that notwithstanding the many moving calls of natural instinct in the child to suck the mother's breast, yet the usual practice is to deny that indulgence till the third day after the birth; by that time, the suppression* of the natural evacuations of the milk, usually brings on a fever, the consequence of which is often fatal to the mother, or puts it out of her power to suckle her child at that time. The sudden swelling of the breasts, which commonly happens about the third day, is another bad consequence of this delay. When the breasts become thus suddenly and greatly distended, a child is not only utterly unable to suck, but, by its cries and struggling, fatigues and heats both itself and the mother: this is another cause which prevents nursing. I believe it was the gentlemen who had the care of the Lying-in Hospital in London, who first had the honour, in this instance, to bring us back to Nature and common sense; and by that means have preserved the lives of thousands of their fellow creatures. They ordered the children to be put to the mother's breast as soon as they shewed a desire for it, which was generally within ten or twelve hours after birth; this rendered the usual dose of physic unnecessary; the milk fever was prevented; the milk flowed gradually and easily into the breasts, which before were apparently empty; and things went on in the natural way. I am sorry, however, to observe, that this practice is not yet become general. If a mother is determined not to nurse her own infant, she should, for her own sake, suckle it at least three or four weeks, and then wean it by degrees from her own breast; in this way, the more immediate danger arising from repelling the milk, is prevented. When a mother does not nurse her own infant, she does open violence to Nature; a violence unknown among the

---

* It is, perhaps, unnecessary to remind our readers, that in publishing the communications of our Correspondents, we by no means wish to be understood, as adopting or approving their opinions. We believe that several opinions contained in this paper, have originated in a want of opportunity to acquire the *latest information*, respecting some of the practices which the writer condemns. Edit.

the inferior animals, whom Nature intended to ſuckle their young; a violence unknown to the moſt barbarous nations, and equally unknown amongſt the moſt poliſhed in the pureſt ages of Greece and Rome.

[ To be continued. ]

